# Young Carers’ Needs and Changing Experiences during an Era of Austerity

**DOI:** 10.3390/ijerph20043418

**Published:** 2023-02-15

**Authors:** Janet L. Warren

**Affiliations:** Department of Social Work and Social Care, University of Bradford, Bradford BD7 1DP, UK; j.warren1@bradford.ac.uk

**Keywords:** young carers, young caregiving, children, informal care, austerity

## Abstract

Many children caring for ill or disabled family members remain ‘hidden’ and ‘invisible’ in our communities. This study is the first to explore patterns of change, over time and throughout austerity, for children with caregiving roles to better understand how their lives differ from those of their non-caregiving peers. A survey of 2154 children, aged 9–18 years in the general population, and a further 21 children, aged 8–18 years and known to be young carers from the same English unitary authority, was conducted to gain an in-depth understanding of children’s perceptions and experiences of what they do to help at home. This study shows that children with caregiving roles remain a distinctive group who assume more domestic and caring responsibilities than their peers, and who also perform many of these activities more frequently than young caregivers in 2001. Approximately 19% of the respondents in the general population showed signs of being in a caring role, double the percentage identified by the author in 2001, 72% of whom were from Black and Minority Ethnic backgrounds. Indicating over time higher levels of unmet needs among parents and other family members who are ill or disabled, these findings have important implications for professional policy, planning and practice across adult and children’s services.

## 1. Introduction

### 1.1. Children’s Caregiving

The extent and nature of children’s informal, unpaid care work within the family has been a growing concern for UK policymakers and practitioners in health, social care and education for over three decades. While some degree of caring and household responsibility is to be valued and encouraged in childhood [1] as a reciprocal part of family life and as a beneficial training ground for good citizenship, the nature and extent of such responsibility need to be congruent with age and levels of physical development and emotional maturity [2] if caregiving is not to become associated with negative outcomes for children’s health, well-being and development. 

Whilst no scientific baseline exists for establishing ‘normal’ expectations of children’s help in running a household, a small body of research from the UK [1,3,4,5], Australia [6], USA [7], sub-Saharan Africa [8], Austria [9] and Switzerland [10] evidences the differences between what young carers do in the family compared with what other groups of children and young people do. Warren’s research in 2001 was the first study to specifically examine the differences between young carers’ lives and those of other children and found that in the UK, what sets young carers apart from their peers is the extent of their caregiving, its nature, the time involved and the outcomes for children’s development, as well as social and economic participation [2,4]. Comparing the caring tasks of ‘known’ young carers with a group of almost 400 children and young people randomly selected from the general population, Warren found that young carers performed a wider range of domestic, emotional, general nursing-type care and intimate care tasks, and spent longer on these activities than other children. 

### 1.2. Hidden Nature of Children’s Caregiving

Over the past two decades, there has been growing recognition of the ‘hidden’ nature of young caring in the UK [2,4,5,11,12]. In 2001, Warren found, unexpectedly, the existence of a ‘hidden’ group of young carers in the general population who had been neither formally recognised in their caring roles nor formally assessed as young carers [4,5]. Approximately one tenth of this randomly selected sample of children were shown to have considerable caring responsibilities, sometimes as substantial and significant as the known young carers in the study, suggesting a hidden group of children within the population who were unlikely to be in receipt of any dedicated support services or interventions. 

Although the UK Census 2011 figures show that 177,918 young carers under the age of 18 provide unpaid care, a percentage increase of 18.7 between 2001 and 2011 [13], the likelihood is that official statistics, undertaken over a decade ago, severely underestimate the true extent of child caregiving [2,3,4,5,11]. Many young carers, and particularly those caring for family members with substance dependency, mental illness or HIV/AIDS, remain hidden from official sight for a host of reasons, including family loyalty, stigma, fear of being taken into care, bullying and not knowing where to go for support [11,14]. This sub-group of young carers, most of whom in Warren’s 2001 survey were from Black and Asian backgrounds, are potentially more vulnerable than ‘known’ young carers in that these children and young people may not have been formally recognised in their caring roles nor formally assessed as young carers and, therefore, are unlikely to be in receipt of services and support. 

### 1.3. Purpose of the Study

This research set out to replicate and expand Warren’s original 2001 study in one southeastern English unitary authority to explore patterns of change for children with caregiving roles, thereby adding to knowledge about the specific needs and changing experiences of young carers over time, including children’s own viewpoints about the definition and perceptions of caregiving, to understand better how their lives differ from other children and young people in the general population who are not known to be looking after someone who is sick, disabled or has other special needs. A secondary aim was to explore children’s views on how the UK Government’s austerity programme (2010–2019) was impacting service provision and children’s unpaid caregiving roles. In response to the 2008 global financial crisis, the UK Coalition and Conservative governments, in office from 2010–2019, introduced a programme of austerity measures to reduce local authority budgets and public expenditure on areas such as social services, welfare benefits and housing subsidies. The introduction of new legislation and amendments to existing legislation including, for example, the Welfare Reform Act 2012 and the Welfare Reform and Work Act 2016, resulted in the capping of a range of welfare benefits such as Child Benefit, Carer’s Allowance, Severe Disablement Allowance, Housing Benefit and Jobseeker’s Support. Under-occupancy penalties, commonly known as the ‘bedroom tax’, were applied to social housing tenants with a spare room who were in receipt of housing benefit, and alongside these welfare changes, the Legal Aid, Sentencing and Punishment of Offenders Act 2012 introduced cuts in legal aid, limiting access to state-funded support in cases relating to welfare benefits, housing, employment, divorce, child contact and clinical negligence. Relative deprivation had risen sharply in the target unitary authority in the interim between both studies. As a suburban area of multiple deprivation, four of the authority’s districts were ranked in the top 10% most deprived neighbourhoods in the UK [15]. Child poverty increased in the authority between 2015 and 2017, placing it within the top 20 local authorities with the highest levels of child poverty across the UK [16]. Unemployment rates were above the regional and national average, and the rate of economic inactivity was higher than average with approximately one-fifth of those who were economically inactive registered as long term sick [17].

## 2. Materials and Methods

### 2.1. Questionnaire

The study integrated both a quantitative and qualitative approach, using a self-completion structured questionnaire to survey 2175 children and young people, aged 9–18 years, to gain an in-depth understanding of their perceptions and experiences of what they do to help at home. 

The original questionnaire used in 2001, which was based on the social survey carried out by the Office for National Statistics [18] and subsequently, developed and tested further by researchers at Nottingham University in 2009 to produce the *Manual of Measures of Caring Activities and Outcomes For Children and Young People* [19], was revised and extended further. The questionnaire employed a combination of both open and closed questions, the purpose being:

- To establish the nature and extent of domestic and caring tasks performed, and children and young people’s perceptions of the help that they provide at home;
- To identify, within the general population, children and young people who may show signs of young caring;
- To explore children and young people’s views about the impact of public sector cuts on service provision and unpaid caregiving roles.

As in the original 2001 survey, a series of closed questions with multiple choice or scaled responses were used to seek specific information about: children and young people’s perception of the nature of the domestic, general, personal and intimate care tasks performed; the levels of responsibility taken for such tasks; and the impacts of helping. To help to identify children in the general population who might be showing signs of being in a caring role, all respondents were invited to identify whether they looked after someone or gave special help to someone at home who was ill, had a disability or other special needs. This question, as in the 2001 survey, was central to establishing children and young people’s viewpoints about definition and perception of their caring roles [5] and helped to categorise respondents into two groups: ‘non-caregivers’ and young people ‘showing signs of caring’. If respondents answered in the affirmative, they were invited to answer two new follow-on questions ‘has anyone assessed your needs?’ and ‘who did the assessment of your needs?’ Staff administering the questionnaire were asked beforehand to identify any known young carers so that their questionnaires could be kept separate from those of the general population sample. 

The questionnaire and consent briefing information were piloted with a small sample of children and young people from a support group for lesbian, gay, bisexual and transgender young people, aged 13–19, located in a metropolitan district council in the north of England, to ensure that the research instrument and information pack were clear and easily understood, inclusive and anti-discriminatory. Taking account of feedback from these young people, adjustments were made to the number of items, the specific wording of some questions and themes, and the range of responses for some items which were not considered to be sufficiently comprehensive. 

### 2.2. Sampling

Using a form of cluster sampling adopted in the original study, 2154 children and young people were randomly selected from educational establishments located in the same districts that had been previously targeted in 2001. Through a lengthy process of negotiation with Head Teachers, Executive Principals, Senior Leadership teams and Family Workers, six educational establishments were identified that were willing to mediate access to a broad sample of mainstream children and young people. This included one sixth form college maintained by the Local Authority (16–19-year-olds), two multi-academy trust (MAT) sponsor-led secondary schools with a sixth form provision (11–18-year-olds), one MAT converter secondary school (11–16-year-olds), one community primary school (4–11-year-olds) and one community junior school (7–11-year-olds), both of which were maintained by the Local Authority. In line with the original study, two year groups (Years 5–6) were chosen in each of the primary and junior schools, as well as the sixth form college (Years 12–13). The remaining schools were asked for the questionnaire to be administered to as many children as possible across each year group, and sufficient hard copies were provided so that every child could be offered the opportunity to complete the questionnaire. Due to an exam period, the questionnaire was administered to Years 7–10 and 12–13 only in one secondary school. 

Four of the six educational establishments’ geographical catchments were located in districts that had been ranked in the top 10% or 10–20% of all Lower Super Output Areas (LSOA) nationally [15], and almost half of students attending the sixth form college lived in the most deprived districts [20]. 

A further 21 children and young people were recruited via a dedicated Young Carers Project and a Young Carers Support Group hosted at the sixth form college, on the basis that they were known to be young carers. This approach to recruitment has been used in several young carer studies [21,22], including the original 2001 study, in which accessing children with caregiving roles had proven particularly difficult. 

### 2.3. Ethical Issues

Ethical approval for the study was obtained from the Humanities, Social and Health Sciences Research Ethics Panel at the University of Bradford and a Mental Health and Emotional Wellbeing Service for Children and Young People Ethics Panel in the locality where the research was being undertaken. Information about the study was distributed in advance of the fieldwork to enable young people and parents of children under 16 years to make an informed decision about participation. This took the form of the following:

- Separate letters and information leaflets to parents and pupils/students;
- Poster displays about the research in each school/college;
- Information events for pupils/students during year group/class assemblies and a separate event for parents;
- Pop-up information stalls in an area familiar to pupils/students.

The Principal Investigator’s contact details were provided in the information letter to the parents of known young carers so that they and their child(ren) could make direct contact to discuss the research.

Participation in the research was both anonymous and voluntary. Staff in each school co-ordinated responses from any children/young people or parents who did not wish their children to participate in the study. Parents and children were advised that if there was a difference of view which could not be mediated, their child could not participate in the study. Young people aged 16–18 with sufficient understanding were considered able to give their full consent to participate in research independently of their parents and guardians/carers [23]. However, college staff were asked to consider seeking parental consent, if appropriate, for particular 16–18-year-olds who may be perceived as vulnerable, and to seek consent from Social Workers for any children and young people up to the age of 18 who were looked after by the Local Authority. 

Respondents were made aware that they did not have to answer any question that they did not feel comfortable answering, they could withdraw at any time and if they stopped completing the questionnaire part of the way through, they would be given the choice about whether their data were removed from the project’s records or whether they were happy to submit their questionnaire. A member of the pastoral care/counselling staff was available to offer specific support and follow up to any participants who needed to talk or access local support services. 

### 2.4. Data Collection

Given the hidden and sensitive nature of young caring, the questionnaire was designed to be completed by children and young people without assistance, but because of the differences in age and ability in a large sample of children and young people, arrangements were made for teachers and support workers, who were attuned to respondents’ individual communication needs, to be available to support the administration of the questionnaires, to ensure that individual children, and particularly those with learning disabilities or communication and sensory impairments, were not excluded or negatively impacted by the process. Such an approach was considered advantageous in the following aspects:

- Drawing on children’s prior experience of similar formats in tests and exam papers;
- Achieving a high level of anonymity;
- Obtaining the views of less confident children more easily;
- Guaranteeing a high response rate [24].

The questionnaires were distributed to pupils in participating secondary schools through the tutor system. In the primary/junior schools, they were distributed by teachers during class. The Principal Investigator administered the questionnaire at the sixth form college in an area frequented by students. The survey was administered to known young carers by a support worker either at their home, school, college, young carers project, or over the telephone, according to the children/young people’s wishes and those of their families. This ensured total anonymity and confidentiality.

### 2.5. Data Analysis

Data collection and analysis occurred in parallel across the two stages of the research. Statistical analysis was undertaken using a quantitative data analysis computer software package (SPSS for Windows ver. 23.0) to generate a wide variety of descriptive and inferential statistics, including frequency, mean and standard deviation. The following statistical tests were used: chi-square (χ2), bi-variate correlation (Cramer’s V coefficient) and Student’s *t*-test as appropriate. The level of statistical significance was set at *p* ≤ 0.05.

Respondents were categorised into three groups to provide quantitative data on the types and levels of domestic and caring tasks undertaken by ‘non-caregiving’ children and young people, and those who showed signs of caring within the home, and these experiences were then compared and contrasted with those of children and young people who were known to adopt caring roles. 

Comparisons were made between the quantitative datasets generated in 2001 and 2017 to explore the specific needs and changing experiences of children and young people.

## 3. Results

Overall, 2175 respondents provided full or partial data that were valid for inclusion in the analysis. Within the general population sample, there were 1135 (52.7%) females, 912 (42.3%) males and 7 (0.3%) gender fluid, transgender and agender (68 respondents (3.2%) preferred not to say, 1 (>0.1%) did not know, and 31 declined to give their gender; *n* = 2154). This was comparable with the 58.7% (222/378) females and 41.3% (156/378) males in the 2001 study. Participants’ ages ranged from 9–18 years, with 72% (1519) of secondary school age (11–15 years), 12.2% (257) aged nine to ten years and 15.8% (334) aged 16–18 (*n* = 2110). The mean average was 13.3 years (SD = 2.1; *n* = 2110) which was slightly higher than that in the 2001 study (12.5 years; SD 2.8; *n* = 378) in which less than half (42.7%) of the participants were aged 11–15 years. The majority of the children and young people were from Asian/Asian British backgrounds (52.4%; 111/2121), with fewer from white (26.5%; 562/2121), Black African/Caribbean/Black British (12.8%; 272/2121) and dual heritage backgrounds (7.4%; 157/2121), compared with the 2001 study, in which three-quarters (74.6%; 282/377) of the sample were White European. Similar proportions of participants as in the 2001 study lived in households in which there were at least two adults (83.7%; 1783/2130 compared with 83.9%; 317/378 in 2001) and at least one of these adults was in full- or part-time employment (95.9%; 1892/1972 compared with 91.8%; 345/376 in 2001). 

The known young carer sample was aged between 8 and 18 years, with the majority (35%; 7/20) falling within the secondary school band aged 11–15 years, compared with 66.7% (8/12) of the respondents in the 2001 study. The mean average age was 13.5 years (SD = 3.2; *n* = 20), compared with 13.8 years in 2001 (SD = 2.4; *n* = 12). The gender division was similar to the 2001 study, with 14 (66.7%) females and 6 (28.6%) males (1 (4.8%) who did not provide their gender; *n* = 21), compared with 8 (66.7%) females and 4 (33.3%) males in 2001 (*n* = 12). Most children (13; 65%) were from white backgrounds, 4 (20%) were Asian/Asian British, 2 (10%) were Black African/Caribbean/Black British and 1 (5%) was dual heritage (*n* = 20). This was similar to 2001 when 7 (58.3%) were white and 5 (41.7%) were dual heritage (mostly white/Asian) (*n* = 12). Fewer known young carers (42.9%; 9/21) were living in lone parent families, or exclusively with their mothers than in 2001 (75%; 9/12) and the majority (71.4%; 15/21) lived in households in which at least one adult was working, usually the young carer’s parent, step-parent, parent’s friend or sibling. This contrasted sharply with the 2001 sample when 66.7% (8/12) of known young carers had lived in households in which no adult was employed—in all cases, these children and young people lived with a lone mother. 

The study provides new knowledge about the specific needs and changing experiences of young carers, what they do to assist in the home, how they feel about what they do and how their lives continue to differ from other children and young people in the general population who are not looking after someone who is sick, disabled or has other special needs. It also uncovers new knowledge about the changing experiences, over time and throughout austerity, of a sub-group of young people in the general population showing signs of caring, whose caring roles and responsibilities continue to remain ‘hidden’. 

### 3.1. How Many ‘Hidden’ Children Showing Signs of Caring?

In total, 392 children and young people (18.7%; *n* = 2091) self-identified as looking after or giving help to someone at home who was ill, had a disability or other special needs, with less than a tenth (8.5%; 178/2091) indicating that they did not know whether they provided such assistance. This finding suggests that the proportion of children and young people showing signs of caring in this southeastern unitary authority had more than doubled since 2001, when just under a tenth (9%; 34/378) of the general population of children and young people self-identified as providing such assistance. A smaller national survey of 925 secondary school pupils from years 7 (aged 11–12 year) and 10 (aged 14–15 years) only, undertaken in different UK locations by the BBC and University of Nottingham in 2018, found that 22% (200/925) of these young people came within the survey’s operational definition of being a young carer [3]. Whilst adopting different methodologies, both pieces of research together provide strong evidence to suggest an increase in the prevalence of children and young people providing care in the UK following a decade of the UK Government’s austerity programme. 

### 3.2. A Profile of the ‘Hidden’ Children Showing Signs of Caring

The gender division of this sub-sample was roughly equal with 187 (48.4%) males, 185 (47.9%) females and 1 agender (0.3%) (13 (3.4%) preferred not to say; *n* = 386). This represented a fall since 2001 in the proportion of females showing signs of caring (58.8%; 20/34) and an increase in the proportion of males (41.2%; 14/34). Over two-fifths (41.2%; 14/34) of participants were aged 11–15 years, the mean average age being 12.8 years (SD = 2.2; *n* = 380), similar to that in the 2001 study (13 years; SD = 2.9; *n* = 34). There was no statistically significant difference between the mean ages of males (12.6; SD= 2.0; *n* = 181) and females who showed signs of caring (13; SD = 2.5; *n* = 180); (t(336.2) = −1.6; *p* = 0.111).

Almost three-quarters (71.9%) of this sub-sample described their origins and background as Black/Black British, Asian/Asian British or dual heritage, the largest minority groups being Asian/Asian British (50.8%; 198/390) and Black African/Caribbean/Black British (12.6%; 49/390) with 27.4% (107/390) white and 8.5% (33/390) dual heritage. Over time, these data represent a 12.9% increase in the proportion of children showing signs of caring from Black and Minority Ethnic (BME) backgrounds [5]. A district of longstanding international migration, this ‘super-diverse’ authority had over 140 nationalities within its population [20], with over 76% of its school pupils from BME backgrounds, more than half (52.4%) of whose first language was not English [25]. 

Almost double (17.2%; 67/390) the proportion of children and young people showing signs of caring lived in a one-parent family, almost exclusively with their mothers (16.2%; 63/390) with 1% living with either their fathers (2/390) or grandmothers (2/390), compared with the 2001 study (8.8%; 3/34) in which this sub-group of young carers lived exclusively with their mothers. Whilst in 2001, a large minority (24.2%; 8/33) of children and young people showing signs of caring lived in households in which no adults were employed, this decreased over time by over two-thirds to approximately 7.3% (25/341). 

Of the 392 children and young people who self-identified as looking after or providing special help to someone at home who was ill, had a disability or other special needs, 357 responded to a new question, not asked in the 2001 survey, about whether they thought their own needs had been assessed. When a UK local authority carries out a young carer’s needs assessment, it is required by The Young Carers (Needs Assessments) Regulations 2015 for the following to be determined:

- The amount, nature and type of care provided.
- The extent to which the care provided is relied upon by the family.
- Whether the care provided is excessive or inappropriate.
- Whether the care impacts the child’s well-being, education and development.
- Whether any of the child’s needs for support could be prevented by providing services to the person cared for or another family member.

Whilst almost a third (32.2%; 115/357) of these children reported that they had received an assessment of their needs, almost a third (30%; 107/357) didn’t know whether their needs had been assessed and strikingly, almost two-fifths (37.8%; 135/357) reported that their needs had not been assessed. If almost a third of this sub-group of children and young people had, indeed, been formally assessed, their schools were unaware of this and were, in consequence, unable to provide appropriate support and guidance to these young people. However, given that 49% (53/108) of these respondents reported that their assessment had been conducted by a family member, such as a parent, grandparent, aunt or sibling, with only 23.1% (25/108) conducted by a Social Worker, 15.7% (17/108) by a Young Carers Project Worker, 4.6% (5/108) by a health care professional such as a Doctor or Health Visitor, 2.8 % (3/108) by a Teacher, 1.9% (2/108) by a Child Minder, 0.9% (1/108) by a Counsellor and 0.9% (1/108) by both a Social Worker and Young Carers Project Worker, it seems likely that many of these children had not received a needs assessment undertaken by an appropriately trained professional with sufficient knowledge and skill to be able to carry out that assessment as defined by The Young Carers (Needs Assessments) Regulations 2015. Overall, these data reveal the continued existence of a distinct group of children and young people within the general population who had been neither formally recognised in their caring roles nor formally assessed as young carers, and who, on the face of it, continued to remain ‘hidden’ or ‘invisible’ in their communities.

### 3.3. What Roles and Responsibilities do Children Undertake?

#### 3.3.1. Domestic Tasks

The study provides insights into the changing nature and extent of domestic work generally undertaken by children and young people during a period of austerity, while also highlighting the extensive range of domestic tasks performed by young people showing signs of caring over and above what other children and young people do. 

Children who did not self-identify as undertaking a caring role (non-caregivers) performed a wider range of domestic tasks in and around the home than they had done in 2001. While they most commonly tidied, dusted and vacuum cleaned their own bedroom, or made light meals such as sandwiches, they were also more likely than they had been in 2001, to tidy, dust and vacuum clean communal areas, lay the table and iron their own clothes regularly, as shown in Table 1 and Table 2. However, beyond this, the level of responsibility assumed by these children and young people for a range of other domestic tasks assumed by young carers and children showing signs of caring was considerably less. Overall, while children and young people in the general population were doing more than in 2001, they continued to spend less time on domestic tasks than ‘caregiving’ children, with just over 60% of those surveyed spending less than five hours per week.

In contrast, the sub-group of children and young people showing signs of caring regularly performed a much wider range of domestic tasks than other children and young people in the general population, as shown in Table 1 and Table 2. In addition to tidying, dusting and vacuum cleaning their own bedroom and communal areas of the home, as well as making light meals, they also regularly helped with lifting and carrying heavy things, decorated rooms, made main meals, shopped for food, washed their own and other people’s clothes, mowed the lawn, weeded the garden and did repairs to the home. They were also more likely than non-caregiving children to spend longer hours (over six hours per week) performing these tasks. With the exception of tidying and dusting their own bedroom (χ2 = 16.502, df = 12, *p* = 0.169), hoovering their own bedroom (χ2 = 13.399, df = 12, *p* = 0.341) and communal areas (χ2 = 20.355, df = 12, *p* = 0.061), and ironing their own clothes (χ2 = 18.835, df = 12, *p* = 0.061), the differences were statistically significant (*p* ≤ 0.001 *p* = 0.034) with over 61% (11/18) of household activities showing a highly statistical significance. Moreover, when comparing the two datasets over time, this sub-group of caregiving children were also performing a much more extensive range of domestic tasks and assuming greater responsibility, performing most tasks more frequently, than in those in the 2001 study.

In contrast, while known young carers were less likely than those in the 2001 study to help with lifting and carrying heavy things, laying the table, ironing their own clothes, making main meals, shopping for food and mowing the lawn, they still continued to undertake a larger range of core domestic tasks (activities needing to be performed most often) than other children and young people in the general population, and were more likely to take full responsibility for these tasks, performing them more frequently and spending longer hours (over six hours) on these tasks each week. As Table 1 and Table 2 show, these tasks included washing up dishes or stacking the dishwasher, making main meals, washing their own and other people’s clothes, and ironing clothes for other people.

#### 3.3.2. General Care and Other Tasks

As in 2001, young carers and children showing signs of caring are still more likely than ‘non-caregiving’ children to regularly undertake a wide range of general and other care tasks including paperwork, dealing with financial matters, taking someone to the doctors or hospital and providing child care, as shown in Table 3 and Table 4.

Data from the new questions asked in the survey show an extended range of tasks that young carers and children showing signs of caring are more likely to perform than ‘non-caregiving’ children, including taking their brothers and sisters to school, working part time to bring in money to the family, and talking with officials such as doctors and the benefits office on behalf of the person they provide care to. The difference was highly significant for all general and other care tasks (*p* ≤ 0.001). As in 2001, these groups of caring children undertook these general care tasks more frequently and spent much longer hours (over 3 h) each week on them than their peers and also spent longer hours performing these tasks than, as a group, they had done in 2001, some spending over 25 h a week.

#### 3.3.3. Personal and Intimate Care

While, historically, young carers have been more likely to regularly undertake a range of personal and intimate care tasks and spend longer periods of time performing these tasks each week than children and young people who do not assume caring roles [2,4,5], the data show a sharp increase over time in the nature and extent of personal and intimate care offered by these children and young people. The young carers interviewed in this study were not only undertaking more personal and intimate care than any other sub-group of children but were also more likely to perform these tasks than young carers in the 2001 study. As Table 5 and Table 6 show, there were considerably more young carers giving medication and injections; changing dressings; helping someone to walk, get upstairs or out of bed; and giving assistance with dressing, washing, bathing, showering and using the toilet than in 2001, and they were also performing these tasks more frequently than they did in 2001.

As in 2001, the research also highlighted a sub-group of caring children and young people showing signs of being in a caring role who, like young carers, performed more tasks of a personal and intimate nature and with greater frequency than non-caregiving children and young people. As Table 5 and Table 6 show, children showing signs of caring and known young carers were much more likely than non-caregiving children and young people to give medication, injections and change dressings; get up during the night or stay up late to look after someone; and give assistance with walking, getting upstairs and getting in and out of bed. They also spent longer hours on personal and intimate care each week than other children and young people in the general population. Less than 15% (191/1298) of children and young people who were not carers spent in excess of three hours per week on personal and intimate care, compared with over half of young carers (57.1%, 12/21) and a third (33.3%, 108/324) of young people showing signs of being in a caring role, some of whom spent over 25 h per week doing so. The difference was highly significant for all personal and intimate care tasks (*p* ≤ 0.001). While this sub-group of caring children and young people were less likely to perform some intimate care tasks, such as giving assistance with washing and dressing, than they had in 2001, they still sometimes performed more personal care tasks and took full responsibility for these tasks more frequently than known young carers or non-caregiving children. For example, as Table 5 and Table 6 show, this group of children were much more likely than their peers to prepare special food or a special diet due to the medical needs of the person they provided care to and to give frequent assistance with eating and drinking.

#### 3.3.4. Emotional Support

The study also highlights the increased levels of emotional support provided by all children and young people during a period of austerity. Overall, Table 7 and Table 8 show that while considerably more non-caregiving children offered emotional support than in 2001 and performed these tasks more frequently than in the past, young carers and young people showing signs of being in a caring role were still more likely than their peers to give emotional support by ‘keeping someone company’ and ‘keeping an eye’ on someone to make sure they were alright. They were also more likely to take full responsibility for these tasks more frequently than non-caregiving children and young people, and spend longer hours giving such support each week than other children and young people in the population.

Just over a tenth (12.9%, 169/1307) of children and young people who were not carers spent an excess of three hours per week providing emotional support, compared with over half of young carers (57.1%, 12/21) and almost a third (30.3%, 98/323) of young people showing signs of being in a caring role, some of whom spent over 25 h per week doing so. Furthermore, Table 7 and Table 8 show that while more children and young people generally were ‘taking someone out’ to see friends or relatives or accompanying them on a walk than in 2001, approximately three-quarters (75.2%, 273/363) of young people showing signs of being in a caring role offered such emotional support compared with just over half (59%, 855/1450) of non-caregiving children and almost half (47.6%, 10/21) of known young carers, and when they offered such support, they also performed this task more frequently than their peers. The difference was highly significant for all emotional support activities (*p* ≤ 0.001).

### 3.4. What Support do Children and Young People Receive?

Overall, the research findings indicated that fewer households in which children and young people had caring responsibilities received Social Work support than in 2001. As Table 9 shows, young carer households were considerably less likely to receive visits from a Social Worker (down almost a third (31%) from 50% (6/12) in 2001 to 19% (4/21), compared with households in which children and young people showed signs of being in a caring role (down just over three percent from 14.7% (5/34) in 2001 to 11.6% (35/301). While these households were slightly more likely to receive home care than in the past, 73% (214/293) of households with children and young people showing signs of caring and 70% (14/20) of young carer households remained unsupported by a professional home care agency, with a further 17.7% (52/293) of young people showing signs of caring and 5% (1/20) of young carers uncertain whether their family received any home care. The study highlights that many of these young people gave assistance with personal and intimate care, including washing, bathing, showering, and using the toilet, without the services or support of a Social Worker, Home Carer, Community or District Nurse. They also undertook responsibility for an extensive range of domestic, general and other care tasks, as well as offered emotional support, without the additional assistance of a Home Care Agency.

### 3.5. Children’s Perceptions of the Extent of Their Help over Time 

The study provides insights into children and young people’s perceptions about the impact of austerity on service provision and unpaid caregiving roles. As Table 10 shows, just over a third (35.4%, 109/308) of young people showing signs of being in a caring role thought that children and young people had to do more to help at home than they had done 15 years earlier, compared with roughly equal but lower proportions of young carers (25%, 5/20) and non-caregivers (24.6%, 314/1273).

While none of the young carers interviewed provided reasons for their response to this question, children in the general population provided a varied range of responses. The primary reasons given by children showing signs of caring reflected their feelings of obligation to family members and strong values centring on the benefits of helping them. While many of the non-caregivers also reported that it was ‘good’ to help at home, a roughly equal number of responses focused on the appropriacy of increased levels of responsibility being commensurate with age and maturity. Only a small minority of all children reported rising costs, financial hardship and a lack of services and support as reasons for why they felt children had to do more 15 years on.

## 4. Discussion

We aimed to recruit a sample of children and young people in the general population from educational establishments located in the same areas targeted previously in the 2001 study so that direct comparisons could be made. However, although the survey conducted was thorough, there were some limitations. First, a few Head Teachers, in their role as gatekeepers, were unwilling to mediate access. The schools selected were, therefore, not representative of all geographical areas surveyed in the original study. Second, while the questionnaire was considered accessible to all respondents based on feedback during the pilot phase, teacher and support worker assistance to those children with special needs, such as learning disabilities, or communication and sensory impairments, may have introduced the potential for bias. Third, the 2001 study highlighted that children who are caregivers are more likely to miss school than non-carers [2]. It is likely, therefore, that the research undercounts the true extent of child caregiving, particularly among the sub-group of children showing signs of caring. Fourth, the young carer sample was recruited via identified educational family workers and a specialist Young Carers Project, and so it may not be representative of the young carer population. Due to cutbacks, the Project was working with young carers with the highest levels of need. Those with lower levels of caring responsibility and need were unlikely to meet service threshold criteria and may or may not have been surveyed as part of the general population sample depending on the geographical locality of their school. Conversely, the need for parents of young carers to give ‘opt-in’ consent will have lowered the response rates and may have biased the sample towards populations with slightly lower levels or specific types of need and caring responsibilities. There may not have been an opportunity for some parents to respond to or contact the Principal Investigator for further discussion about participation. Fifth, estimates of the hours spent on domestic and general tasks, personal and intimate care tasks, and emotional support relied not only on children and young people’s recall but also their ability to assess and quantify time spent on these helping tasks. While these data may be numerically inexact, they do, nevertheless, shed light on children and young people’s perceptions of the levels of responsibility that they assumed.

Overall, the research findings suggest that conventional expectations of children’s help in running a household change during a period of austerity. The survey data suggest that children and young people’s help around the home increased over time with them performing more domestic, emotional and personal care tasks, sometimes more frequently than children and young people did in 2001. However, the lack of choice facing children and young people with caregiving roles and their families means that they remain a distinctive group who spend more time on domestic and caring tasks, and perform these tasks even more frequently than in 2001, more often taking sole responsibility for helping and caring than their peers. Although an explanation for this needs to be tested by further research, the increasing levels of responsibility undertaken by known young carers for a range of core domestic tasks, emotional, general, personal and intimate care tasks during the UK Government’s austerity programme suggest that their ongoing needs may not have been monitored and reviewed sufficiently or regularly, nor appropriate services put in place which might otherwise have reduced the levels of responsibility that they assumed. Continuing to perform these tasks against a backdrop of large-scale funding cuts to benefits, health and social care services may have been the only course of action open to these young carers in the absence of services and support from elsewhere. 

The study also revealed that one in five children and young people in the general population showed signs of being in a caring role, double the ratio reported in the 2001 study. The majority (71.9%) of these hidden caregivers continued to be from Black and Minority Ethnic backgrounds, an increase over time of almost 13%. This sub-group of young carers, most of whom were from Asian and Black African/ Caribbean backgrounds, remained, as in the group in the 2001 study, potentially more vulnerable than known young carers in that many appeared not to have been formally recognised in their caring roles nor their needs appropriately assessed, which might otherwise have reduced the levels of caring responsibility that they assumed. They performed a much wider range of domestic, emotional, personal and intimate care tasks than other children and young people in the general population and spent longer hours supporting family members who were themselves unlikely to be in receipt of dedicated support services or interventions such as Social Work, Community Nursing or Home Care. While this sub-group of caring children and young people were less likely to perform intimate care tasks than they had in 2001, there was still a small range of domestic and caring tasks which they performed more frequently than known young carers. These differences may reflect cultural traditions and expectations, particularly in South Asian communities, of children’s duties to care for older family relatives [26,27]. The changing demographic of respondents from BME backgrounds over time in the general population sample in these two studies may help to explain why assisting at home was perceived by many respondents as ‘good’ and beneficial. However, the myths and racial stereotypes associated with Black and South Asian community family networks caring for ‘their own’, reported at the turn of the century [26], may still permeate service provision. Small pockets of existing research in the UK suggest that young carers and their families from BME communities continue to be more isolated and hidden from services due to a range of barriers, including discrimination and institutional racism, bullying, culturally insensitive services, lack of accessible information, language issues, stigma and fear of agency involvement [11,26,28]. Given that this sub-group of young carers do not constitute a homogenous group, further investigation is needed to capture directly the voices of ‘hidden’ young carers and their families from BME communities who do not access services and support and to understand more fully how and why their needs and experiences continue to differ from known young carers and other children in the general population, in order to inform the future direction of policy and practice for this ‘hidden’ group of caregivers. 

## 5. Conclusions

The Children and Families Act 2014 and the Care Act 2014 provide a duty for UK Local Authorities to identify, assess and provide information and advice to young carers and their families to ensure that no child’s life is negatively impacted as a result of providing care to a family member. Published research highlights the significant challenges posed by stringent cuts in welfare and public services resulting from the UK Government’s austerity programme (2010–2019) and the ‘cost of living crisis’ experienced in the UK since early 2021 [29,30,31,32,33]. Reduced access to local health and social care services and support combined with challenges from the impact of the COVID-19 pandemic continue to have a significant effect on the lives of many individuals and their families, including unprecedented levels of stress, anxiety and financial hardship. 

Whilst this study reports the experiences of children and young people living in one UK urban area with high levels of deprivation, the questions it raises about inequalities in services and support to individuals and their families during an era of austerity have a much wider relevance. The evidence presented challenges all of us, whether we are a service user or carer, a professional in social work and social care, education, community work and health, or a politician, policy maker or researcher, to ask ourselves whether the worsening inequalities in children’s outcomes and life chances resulting from their caregiving roles are acceptable and, if not, what long-term practical solutions should be put in place by the government to ‘level up’ and support these children, young people and their families better.

## Figures and Tables

**Table 1:** Nature of domestic tasks undertaken by non-young carers, young people showing signs of caring and known young carers 2001 and 2016/17.

	2001			2016/17		
Domestic Tasks	% of Non- Caregivers *n* = 334	% of Young People Showing Signs of Caring *n* = 34	% of Known Young Carers *n* = 12	% of Non- Caregivers *n* = 1521	% of Young People Showing Signs of Caring *n* = 392	% of Known Young Carers *n* = 21
Tidy/dust own bedroom	93.7	94.1	91.7	97.6 (1505)	96.9 (381)	90.5
Vacuum clean own bedroom	74.9	73.5	83.3	90.9 (1497)	88.7 (379)	85.7
Make light meals e.g., sandwich	90.7	94.1	91.7	90.8 (1486)	90.1 (375)	85.7
Wash up dishes/stack dishwasher	83.8	67.6	91.7	85.2 (1493)	82.6 (373)	95.2
Help lift/carry heavy things	82.6	91.2	91.7	84.4 (1493)	92.4 (379)	81
Vacuum clean communal areas	70.7	79.4	91.7	84.4 (1484)	86.7 (377)	85.7
Tidy/dust communal areas	70.1	82.4	83.3	84.3 (1480)	87.8 (378)	81
Lay the table	74.3	79.4	75.0	81.5 (1474)	82.8 (372)	66.7
Iron own clothes	53.3	64.7	83.3	66.2 (1499)	62.3 (374)	57.1
Decorate rooms	58.1	61.8	58.8	65 (1490)	76.2 (370)	52.4
Make main meals	53.6	55.9	91.7	59.4 (1479)	67.7 (368)	76.2
Responsible for shopping for food	42.8	47.1	75.0	55.3 (1480)	73.9 (371)	57.1
Wash own clothes	28.4	47.1	50.0	54.1 (1492)	59.3 (376)	76.2
Iron clothes for other people	34.4	58.8	50.0	48.7 (1489)	51.5 (375)	57.1
Weed/look after the garden	44.6	58.8	50.0	43.8 (1487)	54 (372)	55 (20)
Do repairs to the home	28.7	38.2	33.3	40.1 (1490)	61.5 (366)	33.3
Wash clothes for other people	20.4	32.4	41.7	38.1 (1492)	51.6 (372)	66.7
Mow lawn	36.2	47.1	58.3	33.4 (1492)	41.1 (370)	38.1

**Table 2:** Frequency of domestic tasks undertaken by non-caregivers, young people showing signs of caring and known young carers in 2001 and 2016/17.

	2001						2016/17					
Domestic Tasks	% of Non-Caregivers *n* = 334		% of Young People Showing Signs of Caring *n* = 34		% of Known Young Carers *n* = 12		% of Non-Caregivers *n* = 1521		% of Young People Showing Signs of Caring *n* = 392		% of Known Young Carers *n* = 21	
	Always	Mostly	Always	Mostly	Always	Mostly	Always	Mostly	Always	Mostly	Always	Mostly
Tidy/dust own bedroom	18.3	21.6	23.5	23.5	25.0	8.3	32.4	32.6	30.7	33.3	42.9	19
							(1505)		(381)			
Vacuum clean own bedroom	14.4	12.6	17.6	26.5	33.3	0	25.9	24.4	29	25.6	23.8	28.6
							(1497)		(379)			
Make light meals e.g., sandwich	20.4	26.0	26.5	20.6	41.7	41.7	22.9	25.8	33.6	21.9	38.1	23.8
							(1486)		(375)			
Wash up dishes/stack dishwasher	13.8	20.1	23.5	5.9	58.3	25.0	18.3	24	25.2	18.8	38.1	28.6
							(1493)		(373)			
Help lift/carry heavy things	10.8	17.4	23.5	17.6	50.0	8.3	23	21.2	39.1	22.2	33.3	23.8
							(1493)		(379)			
Vacuum clean communal areas	5.1	9.6	17.6	17.6	16.7	8.3	11.7	19.3	17.8	17.5	28.6	14.3
							(1484)		(377)			
Tidy/dust communal areas	4.2	6.0	17.6	5.9	16.7	8.3	9.7	15.7	19.3	19.8	28.6	19
							(1480)		(378)			
Lay the table	10.8	17.4	11.8	23.5	16.7	16.7	15.6	22.5	24.2	21.5	28.6	19
							(1474)		(372)			
Iron own clothes	6.6	8.4	14.7	11.8	33.3	8.3	15.4	12.1	17.9	11.5	33.3	9.5
							(1499)		(374)			
Decorate rooms	1.5	5.4	5.9	5.9	8.3	8.3	6.8	9.5	13.8	16.8	14.3	9.5
							(1490)		(370)			
Make main meals	3.9	6.9	11.8	5.9	16.7	25.0	4.6	7.3	8.2	9.2	9.5	14.3
							(1479)		(368)			
Responsible for shopping for food	1.8	4.2	5.9	2.9	25.0	16.7	4.7	6.5	11.1	14.8	4.8	9.5
							(1480)		(371)			
Wash own clothes	2.7	2.7	5.9	2.9	16.7	0	8.4	6.6	11.2	11.2	23.8	14.3
							(1492)		(376)			
Iron clothes for other people	0.3	3.6	8.8	11.8	8.3	0	4.7	7.3	10.7	10.1	14.3	14.3
							(1489)		(375)			
Weed/look after the garden	1.8	2.4	14.7	5.9	8.3	8.3	3.3	5.2	8.6	9.4	5	20
							(1487)		(372)		(20)	
Do repairs to the home	1.8	0.9	2.9	0	8.3	0	2.8	3.8	6.3	10.1	9.5	0
							(1490)		(366)			
Wash clothes for other people	0.3	1.5	2.9	0	8.3	0	3.4	4	8.6	5.9	19	14.3
							(1492)		(372)			
Mow lawn	1.8	4.8	2.9	14.7	16.7	0	3.7	5.4	6.2	9.2	14.3	9.5
							(1492)		(370)			

**Table 3:** Nature of general and other care tasks undertaken by non-caregivers, young people showing signs of caring and known young carers in 2001 and 2016/17.

	2001			2016/17		
General and Other Care Tasks	% of Non- Caregivers *n* = 334	% of Young People Showing Signs of Caring *n* = 34	% of Known Young Carers *n* = 12	% of Non-Caregivers *n* = 1521	% of Young People Showing Signs of Caring *n* = 392	% of Known Young Carers *n* = 21
Paperwork	44.6	64.7	58.3	48.6 (1458)	65.7 (362)	61.9
Childcare adult nearby	44.6	61.8	41.7	47.6 (1443)	65.3 (352)	57.1
Childcare on own	41.0	58.8	33.3	46.6 (1450)	62.6 (358)	57.1
Take brothers/sisters to school ^🟂^	-	-	-	30.6 (1453)	43.8 (354)	42.9
Financial matters	18.3	29.4	58.3	17 (1458)	34 (359)	28.6
Take someone to doctors/hospital	10.8	47.1	58.3	16.1 (1447)	41.3 (358)	33.3
Work to bring in money ^🟂^	-	-	-	9.1 (1458)	17.6 (357)	19
Talk with officials ^🟂^	-	-	-	9 (1440)	30.3 (347)	33.3

^🟂^ New question in 2016/17 survey.

**Table 4:** Frequency of personal care tasks undertaken by non-caregivers, young people showing signs of caring and known young carers in 2001 and 2016/17.

	2001						2016/17					
Personal Care Tasks	% of Non-Caregivers *n* = 334		% of Young People Showing Signs of Caring *n* = 34		% of Known Young Carers *n* = 12		% of Non-Caregivers *n* = 1521		% of Young People Showing Signs of Caring *n* = 392		% of Known Young Carers *n* = 21	
	Always	Mostly	Always	Mostly	Always	Mostly	Always	Mostly	Always	Mostly	Always	Mostly
Paperwork	1.5	4.5	8.8	5.9	16.7	16.7	3.9	6	12.7	11.9	9.5	9.5
							(1458)		(362)			
Childcare adult nearby	5.4	8.1	5.9	11.8	0	25	8.1	9.9	18.8	16.5	14.3	28.6
							(1443)		(352)			
Childcare on own	4.8	7.5	2.9	17.6	0	0	7.4	8.1	17.6	13.4	14.3	4.8
							(1450)		(358)			
Take brothers/sisters to school ^🟂^	-	-	-	-	-	-	3.7	3.8	8.2	7.3	14.3	4.8
							(1453)		(354)			
Financial matters	0.3	0.3	2.9	0	8.3	16.7	0.9	2	3.1	4.7	4.8	0
							(1458)		(359)			
Take someone to doctors/hospital	1.2	0.6	0	14.7	8.3	8.3	1	1.2	6.4	5	0	23.8
							(1447)		(358)			
Work to bring in money ^🟂^	-	-	-	-	-	-	1.2	1.4	3.9	2.5	0	4.8
							(1458)		(357)			
Talk with officials ^🟂^	-	-	-	-	-	-	0.4	0.8	4.6	3.5	4.8	4.8
							(1440)		(347)			

^🟂^ New question in 2016/17 survey.

**Table 5:** Nature of personal care tasks undertaken by non-caregivers, young people showing signs of caring and known young carers in 2001 and 2016/17.

	2001			2016/17		
Personal Care Tasks	% of Non-Caregivers *n* = 334	% of Young People Showing Signs of Caring *n* = 34	% of Known Young Carers *n* = 12	% of Non-Caregivers *n* = 1521	% of Young People Showing Signs of Caring *n* = 392	% of Known Young Carers *n* = 21
Give medication/injections/change dressings	15.6	52.9	41.7	32 (1473)	72.1 (365)	76.2
Make special food due to medical needs ^🟂^	-	-	-	17 (1464)	52.3 (367)	23.8
Help walk, get upstairs/in and out of bed	9.0	55.9	25.0	17 (1460)	53.6 (366)	57.1
Help eat and drink	4.2	26.5	16.7	15.9 (1463)	44.2 (364)	23.8
Help dress/undress	9.3	50.9	25.0	14.3 (1463)	39.4 (368)	76.2
Get up in night/stay up late to look after ^🟂^	-	-	-	10.5 (1460)	46 (365)	57.1
Help bath/shower	7.2	35.3	16.7	10.4 (1464)	29.1 (364)	57.1
Help wash	5.7	44.1	25.0	10.3 (1462)	30 (363)	57.1
Help use toilet	3.9	23.5	25.0	6.9 (1463)	25.7 (362)	38.1

^🟂^ New question in 2016/17 survey.

**Table 6:** Frequency of personal care tasks undertaken by non-caregivers, young people showing signs of caring and known young carers in 2001 and 2016/17.

	2001						2016/17					
Personal Care Tasks	% of Non-Caregivers *n* = 334		% of Young People Showing Signs of Caring *n* = 34		% of Known Young Carers *n* = 12		% of Non-Caregivers *n* = 1521		% of Young People Showing Signs of Caring *n* = 392		% of Known Young Carers *n* = 21	
	Always	Mostly	Always	Mostly	Always	Mostly	Always	Mostly	Always	Mostly	Always	Mostly
Give medication/injections/change dressings	1.2	0.3	11.8	5.9	0	8.3	3	4.3	15.3	11.8	9.5	14.3
							(1473)		(365)			
Make special food due to medical needs ^🟂^	-	-	-	-	-	-	0.8	1.6	6.5	8.2	4.8	0
							(1464)		(367)			
Help walk, get upstairs/in and out of bed	0	0.6	5.9	11.8	8.3	8.3	1.4	2.9	13.9	11.7	9.5	14.3
							(1460)		(366)			
Help eat and drink	0	0.9	5.9	5.9	0	0	1.7	2.3	8.5	10.7	0	0
							(1463)		(364)			
Help dress/undress	0.3	1.5	11.8	8.8	0	8.3	1.1	2.1	6.3	9.2	9.5	14.3
							(1463)		(368)			
Get up in night/stay up late to look after ^🟂^	-	-	-	-	-	-	0.9	1.1	8.2	6.8	9.5	9.5
							(1460)		(365)			
Help bath/shower	0.6	1.2	5.9	11.8	0	16.7	0.8	1.2	4.1	5.8	9.5	14.3
							(1464)		(364)			
Help wash	0.6	0.3	2.9	17.6	0	25.0	1.2	1.2	3.6	6.9	4.8	19
							(1462)		(363)			
Help use toilet	0.6	0.3	5.9	5.9	0	0	0.5	1	3.3	4.4	9.5	0
							(1463)		(362)			

^🟂^ New question in 2016/17 survey.

**Table 7:** Nature of emotional support given by non-young carers, young people showing signs of caring and known young carers in 2001 and 2016/17.

	2001			2016/17		
Emotional Support	% of Non-Caregivers *n* = 334	% of Young People Showing Signs of Caring *n* = 34	% of Known Young Carers *n* = 12	% of Non-Caregivers *n* = 1521	% of Young People Showing Signs of Caring *n* = 392	% of Known Young Carers *n* = 21
Keep someone company	57.2	85.3	91.7	76.9 (1457)	90.9 (363)	95.2
Keep an eye on someone	56.6	88.2	83.3	74 (1455)	92 (363)	95 (20)
Take someone out	39.5	64.7	33.3	59 (1450)	75.2 (363)	47.6

**Table 8:** Frequency of emotional support undertaken by non-caregivers, young people showing signs of caring and known young carers in 2001 and 2016/17.

	2001						2016/17					
Emotional Support	% of Non-Caregivers *n* = 334		% of Young People Showing Signs of Caring *n* = 34		% of Known Young Carers *n* = 12		% of Non-Caregivers *n* = 1521		% of Young People Showing Signs of Caring *n* = 392		% of Known Young Carers *n* = 21	
	Always	Mostly	Always	Mostly	Always	Mostly	Always	Mostly	Always	Mostly	Always	Mostly
Keep someone company	6.6	10.5	11.8	32.4	25	41.7	18.2	18.1	40.8	24.5	35	35
							(1457)		(363)			
Keep an eye on someone	7.5	12.3	14.7	17.6	41.7	33.3	16.3	18.5	34.2	30.3	23.8	33.3
							(1455)		(363)		(20)	
Take someone out	2.7	6.3	5.9	20.6	8.3	16.7	8.1	11	15.7	16.8	9.5	14.3
							(1450)		(363)			

**Table 9:** Nature of support services received by non-caregiver, young people showing signs of caring and young carer households in 2001 and 2016/17.

	2001			2016/2017		
Services Received at Home	% of Non-Caregivers *n* = 334	% of Young People Showing Signs of Caring *n* = 34	% of Known Young Carers *n* = 12	% of non-Caregivers *n* = 1521	% of Young People Showing Signs of Caring *n* = 392	% of Known Young Carers *n* = 21
Home Carer	0.9	5.9	16.7	1 (1272)	9.2 (293)	25 (20)
Social worker	2.4	14.7	50	2 (1269)	11.6 (301)	19
Community or district nurse	0.9	11.8	25	0.7 (1273)	7.7 (298)	0 (20)

**Table 10:** Children and young people’s perceptions of whether they have to help out more at home than 15 years ago.

Do Children Help Out More than 15 Years Ago?	% of Non-Caregivers *n* = 1273	% of Young People Showing Signs of Caring *n* = 308	% of Known Young Carers *n* = 20
Yes	35.4	25	24.7
No	21.4	30	29.1
Don’t know	43.2	45	46.3

## Data Availability

Not applicable.
